# From hidden hunger to double burden: Bangladesh's urgent need to prioritize diet quality

**DOI:** 10.1016/j.lansea.2025.100673

**Published:** 2025-09-25

**Authors:** Fahmida Tasnim Richi, Safaet Alam

**Affiliations:** a Department of Pharmaceutical Chemistry, Faculty of Pharmacy, University of Dhaka, Dhaka, 1000, Bangladesh; b Department of Pharmacy, University of Asia Pacific, 74/A, Green Road, Farmgate, Dhaka, 1215, Bangladesh; c BCSIR Dhaka Laboratories, Bangladesh Council of Scientific and Industrial Research (BCSIR), Dhaka, 1205, Bangladesh

**Keywords:** Bangladesh, Stunting, Obesity, Non-communicable diseases, Undernutrition, Food security

## Abstract

Bangladesh has achieved notable improvements in nutrition, including declines in undernourishment and stunting. There has been uneven progress, with high rates of stunting and wasting persisting in some areas, such as Sylhet. Food availability has improved, but diet diversity and quality remain low, resulting in chronic micronutrient deficiencies, particularly among women and children. At the same time, rates of overweight, obesity, and diet-related non-communicable diseases (NCDs) are rising, signaling a double burden of malnutrition. Combined with systemic vulnerabilities such as poverty, gender inequality, and climate change, this double burden runs the risk of undoing the progress already achieved. Bangladesh needs to reorient policies to focus on diet quality, increase consumption of micronutrients, and prevent obesity and noncommunicable diseases. The primary recommendations include expanding access to nutrition services, developing climate-resilient food systems, and strengthening nutrition-sensitive governance and policy. Other countries undergoing similar changes can learn valuable lessons from Bangladesh's experience, which underscores the need for an integrated, long-term strategy for nutrition and public health.

**Search strategy and selection criteria**

References for this viewpoint were identified through searches in PubMed, Web of Science, Scopus, Google Scholar, and ResearchGate, using the following search terms: “nutrition transition in Bangladesh”; “stunting prevalence in Bangladesh”; “micronutrient deficiency in Bangladesh”; “overweight and obesity in Bangladesh”; “non-communicable diseases and diet in Bangladesh”; “food security and diet diversity”; and “nutrition-sensitive policies in Bangladesh” to review studies and reports addressing trends in undernutrition, micronutrient deficiencies, obesity, diet quality, and nutrition policies in Bangladesh. In addition, national reports, policy documents, and monitoring data from the Government of Bangladesh, as well as relevant global commitments and frameworks, were included to provide context-specific evidence of high quality.

## Introduction

Bangladesh has made remarkable strides in improving national nutrition indicators over the past two decades. The prevalence of undernourishment (PoU) declined from 13% in 2019–20 to 11.2% in 2022–23, indicating improved food security.^1^ The incidence of stunting among children under five fell from 60% in the mid-1990s to 31% in 2018, and further to 24% by 2022.^1^ These gains reflect the country's sustained investment in multi-sectoral nutrition governance, as operationalized through the National Food and Nutrition Security Policy Plan of Action (2021–2030) and the Second National Plan of Action for Nutrition (2016–2025).

However, reductions in stunting and similar indicators do not necessarily equate to sustained nutritional resilience. Bangladesh is now entering a new phase of the nutrition transition, moving from reducing undernutrition to confronting the dual challenge of undernutrition alongside rising obesity, overweight, and diet-related non-communicable diseases (NCDs). Without urgent policy reorientation to improve diet quality, the country risks eroding its hard-won gains(Panel 1). Bangladesh's experience provides important insights for Southeast Asia, which is undergoing similar shifts in nutritional patterns. This viewpoint is therefore both timely and compelling, offering insights that resonate not only in Bangladesh but also across other countries navigating similar transitions.

Panel 1 **Policy recommendations and regional lessons.**

Bangladesh’s experience offers valuable lessons for South Asia, Southeast Asia, and other regions undergoing similar nutrition transitions. The following policy directions are urgently needed.

**Prioritize diet quality over food quantity**

The nutrition agenda should shift from calorie sufficiency to nutrition adequacy, emphasizing food diversity, nutrient density, and cultural appropriateness.^2^

**Scale up integrated micronutrient strategies**

Home gardening, fortification, adolescent nutrition services, and maternal supplementation should be strengthened and expanded nationwide.^2^

**Address overweight and NCDs with preventive nutrition policies**

Measures should include nutrition labelling, limiting ultra-processed food marketing, expanding school and workplace health initiatives, and investing in youth-oriented food literacy programmes.^1^^,^^4^

**Ensure equity across geography and gender**

Disaggregated data should guide targeted nutrition programming to reach the most vulnerable, especially in regions such as Sylhet and among adolescent girls.^1^^,^^4^

**Build climate-resilient food systems**

Nutrition and food security plans should be integrated with climate adaptation strategies, with special attention to populations in coastal and disaster-prone areas.^2^^,^^4^

**Strengthen multi-sectoral governance**

Nutrition-sensitive interventions across agriculture, education, health, and social protection require coordinated implementation, as outlined in NFNSP.^1^^,^^2^

**Expand school feeding programmes**

School-based nutrition interventions, including meal provision and micronutrient coverage, should be scaled up to maximize the impact to tackle micronutrient deficiency.^2^

**Promote climate-resilient and nutrition-sensitive agriculture**

Diversified cropping systems, homestead food production, and adaptive farming practices should be integrated into agricultural policy and scaled up to safeguard nutrition in the face of climate shocks. This requires addressing structural imbalances in the food system, including overreliance on cereal monocultures, weak incentives for diverse cropping, and insufficient alignment of agriculture with nutrition goals. A shift towards agroecology, integrated farming systems, and investment in local value chains can make nutritious foods more accessible and climate-resilient.^7^

**South–South cooperation and multilateral engagement**

Bangladesh's evolving nutrition challenges also present opportunities for mutual learning and regional collaboration. Many other southern nations are also undergoing such changes and would benefit from focused south–south cooperation for sharing innovations, best practices, and models of implementation.^8^ International organizations, including WHO, FAO, UNICEF, WFP, and IFAD, can provide technical and programmatic support to align national plans with global best practice.^9^^,^^10^

**Aligning with global commitments and frameworks**

Global commitments such as Sustainable Development Goal 2 (Zero Hunger), the UN Food Systems Summit (2021), and the UN Decade of Action on Nutrition (2016–2025) also emphasize improving diet quality, building climate-resilient nutrition systems, and lowering inequities at their core.^7^^,^^11^ Incorporation of these frameworks into national plans strengthens accountability and mobilizes opportunities for advocacy and financing.

## Food system analysis

Bangladesh's food system remains heavily dependent on staple cereal production, especially rice, which contributes to energy adequacy but limits dietary diversity. Dietary energy intake (DEI) from cereals accounted for approximately 58% in 2022–23, down from 64% in 2016, compared with a baseline target of 60% by 2025.^1^ Consumption of fish (from 62.6 g to 67.8 g), vegetables (from 167 g to 201.9 g), and fruit (from 36 g to 95.4 g) increased slightly between 2016 and 2022, though intakes remain under the recommended levels.^1^^,^^2^ Despite recent policy commitments, limited diversification, value-chain inefficiencies, and poor market access constrain the availability and affordability of nutrient-dense foods such as fruits, vegetables, legumes, and animal-source proteins. Strengthening food systems from production to consumption is essential for sustainable nutrition transformation.

## Stunting decline: a success with uneven gains

Stunting has declined nationally, but the progress remains uneven across regions and population groups. In 2022, stunting stood at 24% overall, but reached as high as 34% in the Sylhet Division.^1^ Acute malnutrition has remained stubbornly persistent, with a wasting prevalence of 11% nationally in 2022, exceeding the WHO public health threshold and falling short of Bangladesh's NPAN2 (National Plan of Action for Nutrition, Phase 2) target of 8% by 2025.^1^^,^^3^ Although wasting declined from 16% in 2011 to 14% in 2014 and 9.4% in 2019, the reversal to 11% in 2022 indicates interrupted progress.^1^

Progress in food availability and economic access has not translated equally into improved diet quality. The national PoU figure has improved to 11.2%, yet this aggregate metric conceals serious deficits in the adequacy of diets. The most recent data show that a Minimum Acceptable Diet (MAD) is met by only 29% of children between the ages of 6–23 months.^3^

## Hidden hunger and micronutrient deficiencies

Low dietary diversity contributes directly to persistent vitamin and mineral deficiencies. Micronutrient deficiencies, often termed “hidden hunger”, remain a silent epidemic.^4^ Although less visible than underweight or wasting, micronutrient deficiencies are equally debilitating, impairing cognitive development, lowering immunity, and reducing economic productivity. Recent monitoring indicates that anemia remains prevalent among women of reproductive age, adolescent girls, and children.^3^ Moreover, zinc, vitamin A, folate, and iron deficiency continue to affect millions.^4^

Particularly alarming is the nutritional status of adolescent girls. Poor nutrition during adolescence has long-term intergenerational effects, influencing maternal health, child growth, and economic mobility.^3^^,^^4^ The NPAN2 Monitoring Report highlights widespread anemia and undernutrition in this group, calling for comprehensive adolescent-focused nutrition strategies.^1^^,^^4^

## The rise of overweight and NCDs: a new frontier

Bangladesh now faces a rapidly growing public health challenge: overweight and obesity. Among women, the rate of overweight rose from 11% in 2004 to around 35% by 2021. Among children, it also increased from 1.4% to 2.4% between 2012 and 2019.^4^ These figures signal a worrying trend and mark the early stages of a nutrition transition. This rise is mirrored by the increasing incidence of NCDs such as hypertension, diabetes, and cardiovascular disease. These trends are especially notable in urban and peri-urban areas, where dietary shifts toward ultra-processed, energy-dense, but nutrient-poor foods have accelerated.^3^

Bangladesh is therefore experiencing a double burden of malnutrition, such as undernutrition coexisting with overweight and obesity within the same populations. Bangladesh's food system has yet to adapt to mitigate these emerging risks. Current policies are heavily oriented toward calorie adequacy rather than diet diversity or nutrient balance.^1^^,^^2^ An urgent shift in focus is therefore needed to address these gaps.

## Structural vulnerabilities: inequity, climate, and fragility

Nutrition outcomes in Bangladesh are shaped not only by health and food systems but also by structural vulnerabilities. National progress masks sharp sub-national disparities by region, gender, and income. Regional disparities are pronounced in Sylhet division, for instance, not only records the highest stunting and wasting but also the highest prevalence of maternal chronic energy deficiency (29%).^1^ Women in Sylhet have a lower average body weight compared with other divisions, indicating persistent chronic energy deficiency.^5^ Female-headed households, rural and climate-vulnerable areas, and the poorest income quintiles continue to experience worse nutritional outcomes.^1^

Climate change compounds these challenges. Bangladesh ranks 15th of 163 countries where children are at most risk of climate change.^6^ The government's NFNSP Plan of Action and CIP3 Monitoring Report warn that climate impacts coupled with COVID-19 disruptions and geopolitical price shocks such as the Russia–Ukraine war, have already exposed the fragility of food systems in Bangladesh.^1^^,^^4^ These vulnerabilities are not abstract risks; they are actively exacerbating both undernutrition and the growing burden of overweight, obesity, and NCDs, particularly in low-income households.

## Conclusion

Bangladesh's achievements in reducing undernutrition hold important lessons for low- and middle-income countries. However, the changing nutrition landscape requires a broader definition of success amid persistent micronutrient deficiencies and rising overweight and diet-related NCDs across age and income groups. Conventional markers such as declining stunting are insufficient alone to demonstrate sustained nutritional progress. The country stands at a critical juncture where it must transition from calorie-focused approaches to strategies that promote diet quality, equity, and resilience. Without this shift, earlier gains may be undermined, and the growing double burden of malnutrition could compromise both health and development outcomes. Building inclusive, nutrition-sensitive food systems is therefore not optional; it is the next frontier for public health.

## Contributors

**Fahmida Tasnim Richi**: Visualization, Conceptualization, Data Curation, writing—original draft, and writing—review & editing. **Safaet Alam:** Supervision, Project administration, Conceptualization, writing—original draft, and writing—review & editing. All authors have read and approved the final manuscript.

## Data sharing statement

The manuscript has used publicly available data.

## Declaration of interests

We declare no competing interests.

## Appendix A Supplementary data

In Bengali
