# Supplementary material for: From hidden hunger to double burden: Bangladesh's urgent need to prioritize diet quality
Source: Lancet Reg Health Southeast Asia. 2025 Sep 25;41:100673. doi: 10.1016/j.lansea.2025.100673 (PMC12509728; doi:10.1016/j.lansea.2025.100673)
Supplement: In Bengali [file mmc1.docx]

*This translation in Bengali was submitted by the authors and we reproduce it as supplied. It has not been peer reviewed. Our editorial processes have only been applied to the original abstract in English, which should serve as reference for this manuscript*

বাংলাদেশ পুষ্টি উন্নয়নে গত দুই দশকে অনেক অগ্রগতি করেছে। খর্বকায়তা ও অপুষ্টির হার উল্লেখযোগ্যভাবে কমেছে, এবং খাদ্যপ্রাপ্যতা বেড়েছে। তবে এই অগ্রগতি এখনও সবার মাঝে সমভাবে পৌঁছেনি। যেমন, সিলেট অঞ্চলে এখনো খর্বকায়তা ও চরম অপুষ্টির হার বেশি দেখা যায়।

একদিকে যেমন দেশে খাবারের প্রাপ্যতা বেড়েছে, অন্যদিকে খাবারের গুণমান ও বৈচিত্র্যের ঘাটতি অদ্যাবধি রয়ে গেছে। এর ফলে নারী ও শিশুদের মধ্যে মাইক্রোনিউট্রিয়েন্ট ঘাটতি বা পুষ্টির গোপন অভাব একটি বড় সমস্যা হয়ে বিদ্যমান। একই সঙ্গে বাড়ছে অতিরিক্ত ওজন, স্থূলতা ও খাদ্যসংক্রান্ত অসংক্রামক রোগ, যা বাংলাদেশে পুষ্টি সমস্যাকে একটি ‘দ্বৈত বোঝা’-তে রূপান্তর করছে। দারিদ্র্য, লিঙ্গ বৈষম্য ও জলবায়ু পরিবর্তনের মতো কাঠামোগত দুর্বলতা এই সংকটকে আরও গভীর করছে। এই পরিস্থিতি সামাল দিতে হলে বাংলাদেশকে এখনই নীতিগতভাবে পরিবর্তন আনতে হবে এবং খাদ্যের পুষ্টিমান, বৈচিত্র্য এবং প্রাপ্তিযোগ্যতার উপর জোর দিতে হবে। পাশাপাশি, মাইক্রোনিউট্রিয়েন্ট গ্রহণ বাড়ানো এবং স্থূলতা ও অসংক্রামক রোগ প্রতিরোধে কার্যকর ব্যবস্থা নেওয়াও জরুরি।

এই ভিউপয়েন্টে আমরা যে সুপারিশগুলো দিয়েছি, তার মধ্যে রয়েছে পুষ্টিকর খাবার প্রাপ্তির সুযোগ বাড়ানো, জলবায়ু সহনশীল খাদ্যব্যবস্থা গড়ে তোলা, এবং স্বাস্থ্য, কৃষি ও সামাজিক সুরক্ষার মধ্যে সমন্বয় তৈরি করে একটি টেকসই ও পুষ্টিকেন্দ্রিক নীতি প্রণয়ন। বাংলাদেশের অভিজ্ঞতা শুধু নিজ দেশের জন্য নয়, একই ধরনের পরিবর্তনের মধ্য দিয়ে যাওয়া অন্যান্য দেশের জন্যও গুরুত্বপূর্ণ শিক্ষা হিসেবে কাজ করতে পারে।
